# *Boesenbergia pandurata* application in Goldfish ( *Cyprinus carpio*) Feed to Enhancing Fish Growth, Immunity System, and Resistance to Bacterial Infection

**DOI:** 10.12688/f1000research.52889.1

**Published:** 2021-08-06

**Authors:** Esti Handayani Hardi, Gina Saptiani, Rudi Agung Nugroho, fadlul Rahman, Sulistyawati Sulistyawati, Widyaningsih Rahayu, Ali Supriansyah, Irawan Wijaya Kusuma

**Affiliations:** 1 Microbiology environmental Laboratory, Faculty of Fisheries and Marine Science, Mulawarman University, Samarinda/East Kalimantan, Indonesia, 75123, Indonesia; 2 Research Center of Medicine and Cosmetic from Tropical Rainforest Resources PUI-PT OKTAL, Mulawarman University, Samarinda/East Kalimantan, Indonesia, 75123, Indonesia; 3 Department of Biology,Faculty of Mathematics and Natural Sciences, Mulawarman University, Samarinda/East Kalimantan, Indonesia, Indonesia; 4 Forestry, Mulawarman University, Samarinda, East Kalimantan, 75123, Indonesia

**Keywords:** Boesenbergia pandurata, Cyprinus carpio, Phytobiotics, Aeromonas hydrophila, Pseudomonas fluorescens.

## Abstract

**Background:** This study investigated how the inclusion of Boesenbergia pandurata extract (BPE) in goldfish feed affects fish growth, immunity, and resistance to infection by Aeromonas hydrophila and Pseudomonas fluorescens.

**Methods:** Four fish feeds, were prepared by adding BPE at the concentrations of 0 (control), 2, 4, and 6 g kg-1, respectively, and 120 goldfish (Cyprinus carpio; initial weight 5 g) were separated into 12 boxes and fed with specific pellets and examined thrice. The experiment lasted 12 weeks, beginning with the different feeds, fish growth was measured at Weeks 4 and 8 after the feeding period. Moreover, a challenge test with pathogen bacteria to assay disease resistance was administered at Week 8 after the feeding period, and the survival rate and relative percentage of survival were quantified at Week 12.

**Results**: At Week 8, the goldfish that were fed BPE-containing feeds were significantly heavier than the fish that received the control feed (pellet without BPE), and the highest weight gain, reaching 72.44 g, was obtained with Pellet 3; accordingly, the specific growth rate after BPE treatment (5.7%) was higher than that after control treatment. Conversely, the feed conversion ratio in the control group, 2.03, was higher than the ratios in the BPE groups, which were decreased to 0.55–0.90. Lastly, BPE treatment consistently enhanced the immunity parameters of goldfish (relative to control treatment) at weeks 4 and 8, and following BPE treatment, the rate of resistance against bacterial infection, 68.3%–77.0%, was higher than that after control treatment.

**Conclusions:** BPE addition in goldfish feed clearly produces a positive effect by enhancing fish growth, immunity, and resistance to infection by pathogenic bacteria, and 4 g kg-1 is the optimal BPE concentration in feed prepared for goldfish.

## Introduction

Over the past few years, the use of antibiotics in aquaculture has attracted considerable research attention, and enhanced public awareness of the health and safety of fishery products has led to the insistence that all involved parties concurrently prioritize quality assurance and food safety in aquaculture production, whether for export purposes or domestic consumption. ^1^ Moreover, increasing innovation in aquaculture methods, particularly in the technology used, has been accompanied by a drastic increase in the production. Antibiotics application in aquaculture have been identified as residual materials in fish products and have emerged as the main reason for the frequent rejection of fish products. ^2^ Conversely, the use of plant extracts as one of the ingredients in fish feed to stimulate fish growth and immunity is highly recommended because the extracts produce no resistance effects as residual wastes nor pose any threat to the environment. ^3^^–^^9^

In aquaculture, the use of fish feed prepared from plant extracts offers certain benefits, such as stimulation of growth and immunity, enhancement of digestion and absorption, and resistance to diseases, and also allows for effective control of water quality. ^10^^–^^20^ Notably, all reported single extracts of fingerroot ( *Boesenbergia pandurata*) have been found to exert a strong antibacterial effect (80% inhibition) against *Aeromonas hydrophila* and *Pseudomonas fluorescens* both *in vitro* and *in vivo*, and fish feed containing plant extracts as an additive has been widely developed in aquaculture. ^21^^–^^28^ Moreover, addition of garlic in fish feed increased fish immunity, ^26^ and inclusion of thymol carvacrol effectively enhanced the growth and health of rainbow trout fries. ^29^ This study comprehensively describes the effectiveness of the inclusion of *B. pandurata* (crude) extract (BPE) in fish feed to stimulate growth, immunity, and resistance to *A. hydrophila* and *P. fluorescens* infection in goldfish ( *Cyprinus carpio*).

## Methods

### Aquarium and goldfish

Twelve aquariums (46 × 36 × 25.6 cm ^3^) was used in this research with 25 L of water for 10 fish. There were four groups of different pellets and triplicates with the extracts added and a control. Each group used 10 goldfish, the fish were obtained from Rama Jaya Mahakam Company’s hatchery in Kutai Kartanegara regency, East Borneo, Indonesia, totalling 120 fish in this experiment.

The fish sample was collected using a fish sorting bucket of size 8 cm, fish that escaped from the 8 cm fish sorting bucket were collected. The fish species was goldfish ( *Cyprinus carpio*), the fish sex was mixed between male and female, the developmental stage was larva size 8–9 cm, and the initial weight range was 5 ± 0.6 g.

Before the experiment, the goldfish were adapted to the natural environment for seven days and the fish were provided *ad libitum* access to commercial feed twice a day (at 8.00 a.m. and 4.00 p.m.). Moreover, the fish were first tested for infection by *Aeromonas* and *Pseudomonas* by incubating their isolated liver and kidneys with GSP (Himedia ^®^) media; if the bacteria did not grow the fish were considered safe for use in the experiment, whereas if bacterial growth was constantly detected, the fish were soaked in 30% formalin for five minutes and the treatment was repeated for seven days until they were free from the bacteria, *Aeromonas* and *Pseudomonas.*

### Boesenbergia pandurata (BPE) preparation

The method of Hardi *et al*. ^30^ was used for *B. pandurata* extraction; the rhizome was cleaned to remove soil and then minced into pieces (0.3–0.5 cm) by using a chopper, and the chopped fingerroot was dried at 40–45 °C for 48 h in an oven. The dried fingerroot was continuously blended and soaked in 96% ethanol for 48–72 h at a 1:10 ratio (i.e. 1 kg of fingerroot powder was soaked in 10 L of ethanol), and the process was continued to extraction for 24 h until the BPE was obtained with a viscosity of 10–11.

### Composition of goldfish feed

BPE was used in goldfish feed as per the method of Hoseinifar *et al*., ^31^ with the following four feed pellets being applied as treatments:

Pellet 1 (control diets with BPE 0 g kg ^−1^ fish feed).

Pellet 2 (supplemented-control diets with 2 g kg ^−1^ fish feed of *B. pandurata*).

Pellet 3 (supplemented-control diets with 4 g kg ^−1^ fish feed of *B. pandurata*).

Pellet 4 (supplemented-control diets with 6 g kg ^−1^ fish feed of *B. pandurata*).

Goldfish feed was formulated as shown in Table 1.

**Table 1.** Formulated composition of fish feed (g kg ^−1^).

Ingredients	Pellet 1	Pellet 2	Pellet 3	Pellet 4
Shrimp flour	400	400	400	400
Wheat flour	265	265	265	265
Soybean flour	135	135	135	135
Soybean oil	60	60	60	60
Fish oil	60	60	60	60
Mineral premix	30	30	30	30
Vitamin premix	20	20	20	20
Binder	20	20	20	20
vitamin C	10	8	6	4
BPE	0	2	4	6

### Bacteria pathogen and challenges

The bacteria pathogen for challenges were *A. hydrophila* (EA-01) and *P. fluorescens* (EP-02) combination bacteria with bacterial density of 10 ^5^ CFU mL ^−1^ each bacteria and injection of as much as 0.1 mL were given to each fish. The bacteria were cultured in TSB (Merck ^®^) medium for 24 h in 28–30 °C. Suspense bacteria was collected and centrifuged for 15 minutes in 7000 rpm and bacteria pellet was washing with sterile water twice, and then the bacteria suspense was counting density using TPC to measure 10 ^5^ CFU mL ^−1^, and bacteria had been properly prepared following the methods of Hardi *et al*. (2016).

Challenge tests were carried out at week eight after feeding with different formulations, and mortality observations were checked from 24 hours after the first injection until week 12. The rate of resistance against both bacteria was measured using RPS (Amend, 1981). Eventually, the rate of protection against pathogen bacteria was also measured at week 12.

### Water quality measurement

Three parameters of water quality—temperature, pH, and dissolved oxygen (DO)—were measured (twice daily, in the morning and evening) using a multi-parameter checker, whereas total ammonia nitrogen was measured using a spectrophotometer.

### Examination of immunological parameters

Immunological parameters were evaluated by quantifying total leukocyte (TL) using cells mm ^−3^ numbers and by measuring lysozyme activity (LA) according to the method of Parry *et al*. ^32^ (with the results expressed using the unit μg mL ^−1^). Subsequently, phagocytosis activity (“index phagocytic,” IP) the results expressed using percentage and respiratory burst activity (RBA) were examined as per the method of Van Doan *et al*., ^33^ with a few modifications.

### Fish growth

Goldfish growth was measured according to the method of Hoseinifar *et al*. ^34^ at Weeks 4 and 8 after the feeding period; growth was measured in terms of the following criteria: weight gain (WG), specific growth rate (SGR), and feed conversion ratio (FCR). These data were collected at 8 weeks after the feeding period:

WG = final weight (g) – initial weight (g);

SGR (%) = 100 × (ln final weight – ln initial weight)/duration of experiment;

FCR = feed offered (dry weight)/weight gain (wet weight).

### Challenge test

The challenge test was administered by using *A. hydrophila* (EA-01) and *P. fluorescens* (EP-02); the bacteria were appropriately prepared as per the method of Hardi *et al*. ^6^^,^^35^ The test was administered at Week 8, with 10 goldfish being exposed to a specific treatment; the fish were infected with the combined bacteria by means of intermuscular injection (0.1 mL each fish) of 10 ^5^ CFU mL ^−1^ of the bacteria at a 1:1 ratio. Subsequently, fish mortality was monitored from 24 h after the first injection until week 12. The rate of resistance toward both bacteria was measured by using the relative percentage of survival (RPS) value, as defined in the Amend (1981) method. Lastly, the rate of protection against infection with the pathogenic bacteria was measured at Week 12.

SR (%) = (final fish number/initial fish number) × 100;

RPS = 1 − (test mortality/control mortality) × 100.

### Statistical analysis

The obtained data were analyzed for statistical significance by using MINITAB ^®^ 17 computer program (Minitab, RRID:SCR_014483), followed by the DUNCAN test. The average scores calculated were considered significantly different at *P* < 0.05.

## Results

### Growth performance

Goldfish growth performance was measured at Weeks 4 and 8 after the feeding period. At Week 4, WG and SGR were significantly higher ( *P* < 0.05) after all BPE treatments than after the control treatment (no BPE) ( Table 2). The highest SGR and WG were recorded in the case of the goldfish that received fish feed containing BPE at 4 g kg ^−1^, and these values at Week 8 were considerably different from those measured for goldfish exposed to the control and others treatments.

**Table 2.** Growth performance (WG, SGR, FCR) at Weeks 4 and 8 of *Cyprinus carpio* treated with *Boesenbergia pandurata* extract (BPE).

Parameters	Week	Pellet 1	Pellet 2	Pellet 3	Pellet 4
**WG (g)**	**4**	10.30 ± 0.22 ^a^	19.75 ± 0.2 ^b^	25.99 ± 0.4 ^b^	20.40 ± 0.1 ^b^
	**8**	19.67 ± 0.09 ^b^	47.32 ± 0.02 ^c^	72.44 ± 0.01 ^d^	44.67 ± 0.02 ^c^
**SGR (g)**	**4**	3.99 ± 0.02 ^a^	5.71 ± 0.01 ^b^	7.19 ± 0.01 ^c^	6.46 ± 0.04 ^b^
	**8**	5.70 ± 0.02 ^b^	8.39 ± 0.04 ^c^	10.54 ± 0.03 ^d^	8.92 ± 0.02 ^c^
**FCR**	**4**	2.33 ± 0.11 ^a^	1.22 ± 0.08 ^b^	0.92 ± 0.09 ^c^	1.18 ± 0.1 ^b^
	**8**	2.03 ± 0.06 ^a^	0.85 ± 0.11 ^c^	0.55 ± 0.05 ^d^	0.90 ± 0.1 ^c^

WG = weight gain; SGR = specific growth rate; FCR = feed conversion ratio.

At Week 4, goldfish exposed to the control treatment grew by SGR 3.99–5.70 g; by contrast, treatment with BPE drastically enhanced growth, by 7.19–10.54 g in the case of the feed containing 4 g kg ^−1^ BPE, and this was 2- or even 3-fold higher than that with the control treatment ( Table 2). Moreover, consistent results were obtained at Week 8 after the third fish feeding, with the growth doubling relative to the initial weight and being markedly distinct from that measured after the control treatment. Furthermore, besides growth, feed efficiency also increased, as demonstrated by the FCR increase (relative to control) being substantially lower (0.55) at Week 8 in the case of goldfish that were fed Pellet 3 (BPE at 4 g kg ^−1^), and this FCR value was also significantly different from those calculated after treatment with BPE at the two other concentrations (2 and 6 g kg ^−1^), which produced roughly equal effects.

### Immunological parameters

Next, immunological parameters were measured at Weeks 4 and 8 after the feeding period ( Table 3). Activity of Lysozymes (LA) in addition to control of feed-fish in BPE was significantly higher ( *P* < 0.05) ( Table 3). Compared with other formulas, the highest value was recorded in fish fed pellet 3 (4 g kg ^−1^). No significant difference ( *P* > 0.05) between fish fed 2 and 6 g kg ^−1^ has been observed ( Table 3). Similarly, in additional groups the activity of index phagocytic (PI), compared with the control of fed fish, was significantly higher ( *P* < 0.05) ( Table 3). Fish fed dietary pellets 3 showed the highest values ( Table 3). In relation to the activity of respiratory burst (RBA), fish supplemented diets ( *P* < 0.05) were significantly higher than the control. No significant difference ( *P* > 0.05) between pellet 2 and pellet 4 was however observed. In comparison to controls after 8 weeks of feeding, and 12 weeks after challenges, significant ( *P* < 0.05) differences in total leukocyte (TL) activity were observes in fish-feed supplements ( Table 3).

**Table 3.** Immunological parameters (RBA, IP, LA, and TL) at Weeks 4 and 8 of goldfish treated with *Boesenbergia pandurata* extract (BPE).

Parameters	Week	Pellet 1	Pellet 2	Pellet 3	Pellet 4
**IP (%)**	**4**	20.7 ± 0.58 ^a^	53.7 ± 0.71 ^b^	64.9 ± 1,21 ^b^	55.4 ± 0.4 ^b^
	**8**	24.9 ± 0.4 ^b^	61.3 ± 0.67 ^c^	68.4 ± 0.51 ^d^	64.7 ± 0.75 ^c^
**LA (μg mL** ^**−1**^ **)**	**4**	3.9 ± 0.15 ^a^	5.7 ± 0.08 ^b^	6.2 ± 0.15 ^c^	6.0 ± 0.21 ^b^
	**8**	3.9 ± 0.1 ^b^	5.8 ± 0.05 ^c^	6.54 ± 0.06 ^d^	6.0 ± 0.1 ^c^
**TL (10** ^**4**^ **) (cell mm** ^**−3**^ **)**	**4**	2.4 ± 0.12 ^a^	5.22 ± 0.1 ^b^	7.92 ± 0.15 ^c^	5.81 ± 0.1 ^b^
	**8**	2.6 ± 0.1 ^a^	6.85 ± 0.1 ^c^	8.55 ± 0.1 ^d^	6.90 ± 0.1 ^c^
**RBA (OD)**	**4**	0.3 ± 0.06 ^a^	0.6 ± 0.03 ^b^	0.8 ± 0.06 ^b^	0.7 ± 0.06 ^b^
	**8**	0.5 ± 0.03 ^b^	0.8 ± 0.10 ^c^	1.1 ± 0.12 ^d^	0.9 ± 0.06 ^c^

RBA = Respiratory burst activity; IP = Index phagocytic; LA = Lysozyme activity; TL = Total leukocyte.

### Challenge test

The rate of survival and death and RPS were measured at Week 12 after completion of the challenge test. Unexpectedly, the results showed that treatment with BPE at all concentrations markedly increased the RPS, by >60%, relative to control, although BPE at 4 g kg ^−1^ provided the maximal protection (99.56%) against *A. hydrophila* and *P. fluorescens* infection ( Table 4).

**Table 4.** Rate of survival, death and relative percentage of survival of goldfish at Week 12 after challenge test against *A. hydrophila* and *P. fluorescens* bacterial infection.

Parameters	Pellet 1	Pellet 2	Pellet 3	Pellet 4
**SR (%)**	17	68.3	77	72
**Mortality (%)**	82.3	31.7	23	28
**RPS (%)**		62	72	66

The results of the experiment examining protection against pathogenic bacteria showed a significant increase in goldfish disease resistance, amounting to 68.3–77.0% following all BPE treatments, although no significant difference was measured between the distinct concentrations of BPE ( *P* > 0.05); moreover, the highest RPS value (77.0%) was obtained with BPE used at 4 g kg ^−1^ in the fish feed ( Table 4). In conclusion, relative to Pellet 1, which did not contain BPE, all other pellets drastically increased the rate of survival after pathogenic bacterial infection.

### Water quality

No significant differences were present in the quality of water in the goldfish aquaculture media when BPE was included in fish feed. The temperature was set at 29 ± 0.2 °C, the DO was 7.6 ± 0.6 mg L ^−1^, the pH range was 7.2 ± 0.5, and the total ammonia nitrogen was 0.69 ± 0.24 mg L ^−1^.

## Discussion

Prebiotics prepared from plant extracts have been widely used in aquacultures. The results have shown that plant extracts added to feed enhance fish growth, ^36^^,^^37^ maximize immunity, ^38^ and strengthen disease resistance and thus reduce infection by pathogenic bacteria. ^39^^–^^43^

In this study, we aimed to evaluate how BPE inclusion in fish feed affects goldfish growth, immunity, and disease resistance (i.e. resistance against infection by the bacteria *A. hydrophila* and *P. fluorescens*). The results comprehensively showed that BPE addition in feed exerted the positive effects of enhancing fish growth and strengthening the immune system. Similar results were reported by Hoseinifar *et al*. ^31^^,^^34^^,^^41^ and Carbone & Faggio: ^44^ Addition of the extract of medlar leaf ( *Mespilus germanica*) consistently produced a large impact, with markedly enhanced performance being recorded in terms of growth, skin mucus levels, and serum concentrations of immune-response markers. Our study also showed increased growth of goldfish, particularly at Week 8, after consumption of feed containing BPE at 2, 4, and 6 g kg ^−1^. Thus, BPE served as a growth-stimulating additive for the goldfish aquaculture here. Plant extracts included in fish feed have been reported to markedly increase WG, SGR, protein efficiency ratio, energy retention, feed efficiency, and protein retention. ^15^^,^^45^^–^^47^ Moreover, champignon ( *Agaricus bisporus*) powder extract included in fish feed effectively enhanced growth and acted as an immunostimulant in the case of goldfish fries. ^47^ Our results here indicate that the growth, immune response, and disease resistance of goldfish were strongly influenced by the immunomodulatory effect of BPE.

In previous studies, BPE use at 400–900 ppm successfully strengthened the immune system and enhanced the disease resistance of Nile tilapia toward infection by *A. hydrophila* and *P. fluorescens*. ^10^^,^^48^ Moreover, flavonoid and levamisole addition in feed potently intensified the antigen-phagocytosing effect of monocytes and macrophages, ^45^ and inclusion of BPE alone or together with other extracts boosted leucocyte numbers and consistently accelerated pathogen elimination inside the body of Nile tilapia. ^4^^,^^6^^,^^11^ Subsequently, BPE-containing vaccines were also found to increase the antibody levels and phagocytic index in Nile tilapia to enhance the immune system and produce accelerated and strengthened resistance against infection by pathogenic bacteria. ^4^ An enhancement of monocyte and macrophage function in pathogen elimination, mucosal immune response, growth, and gene transcription is generally observed in fish that are fed plant-extract-containing fish feed formulated with peptin, oligosaccharides, and flavonoids, ^49^^,^^50^ and the use of combinations of plant extracts in aquaculture is also well established. Moreover, addition of *Ferula assafoetida* extract to fish feed was shown to successfully enhance nonspecific immune-system response and growth in carp fish. ^51^ The increased growth caused by fish feed-efficiency enhancement and FCR reduction in BPE-fed goldfish occurred because of the positive physiological impact that carbohydrates (oligosaccharides) and the essential nutrient pectin produced on the digestive system by reducing glucose absorbance ^52^ and postponing gastric emptiness. ^53^ Ho *et al*. ^54^ and Naqash *et al*. ^55^ reported that pectin and its derivatives are components that can potentially be used as prebiotics for aquaculture.

## Conclusion

BPE addition in fish feed provided to goldfish markedly enhances fish growth, feed efficiency, FCR, immunity, and resistance against infection by the bacteria *A. hydrophila* and *P. fluorescens.* Moreover, inclusion of 4 g kg ^−1^ BPE in the feed more strongly affects the aforementioned parameters than does BPE added at other concentrations.

## Ethical approval

The Commission of Ethical Research for Health, Medical Faculty of Mulawarman University, approved this study with the number LOA 04/KEPK-FK/1/2020. The application of *B. pandurata, S. ferox*, and *Z. Zerumbet* in freshwater fish feed to improve fish growth, immune system, and resistance to bacterial infection is the research theme. Esti Handayani Hardi of Mulawarman University's Faculty of Fisheries and Marine Science chaired this study. This study lasted six months (from January to June 2020). For a period of 12 weeks, a feed composition with extracts was tested to see how well the fish grew, how well their immune systems worked, and how well they were protected from infections.

## Data availability

### Underlying data

Open Science Framework, OSF 2021: Underlying data for ‘ *Boesenbergia pandurata* application in goldfish ( *Cyprinus carpio*) feed to enhance fish growth, immunity, and resistance to bacterial infection’. https://doi.org/10.17605/OSF.IO/827EN. ^56^

This project contains the following underlying data:

• Raw data of the growth Performa (Weight gain)
• Raw data of the growth Performa (Specific growth rate)
• Raw data of the growth Performa (Feed conversion ratio)
• Raw data of the Immunological Parameters (Index phagocytic)
• Raw data of the Immunological parameters (Total leukocyte)
• Raw data of the Immunological parameters (Lysozyme activity)
• Raw data of the Immunological parameters (Respiratory burst activity)
• Raw data of the Survival Rate

Data are available under the terms of the Creative Commons Zero “No rights reserved” data waiver (CC0 1.0 Public domain dedication).
