# Supplementary material for: Boesenbergia pandurata application in Goldfish ( Cyprinus carpio) Feed to Enhancing Fish Growth, Immunity System, and Resistance to Bacterial Infection
Source: F1000Res. 2021 Nov 26;10:766. Originally published 2021 Aug 6. [Version 2] doi: 10.12688/f1000research.52889.2 (PMC8667009; doi:10.12688/f1000research.52889.2)
Supplement: Peer review history

# Peer review: Reviewer response for version 2

**DOI:** 10.5256/f1000research.79656.r101240

**Published:** 2021-12-07

**Authors:** Angela Mariana Lusiastuti

**Affiliations:** 1 Research Institute for Freshwater Aquaculture and Fisheries Extension (RIFAFE), Bogor, Indonesia

The revisions are appropriate and sufficient.

Is the work clearly and accurately presented and does it cite the current literature?

Yes

If applicable, is the statistical analysis and its interpretation appropriate?

Yes

Are all the source data underlying the results available to ensure full reproducibility?

Yes

Is the study design appropriate and is the work technically sound?

Yes

Are the conclusions drawn adequately supported by the results?

Yes

Are sufficient details of methods and analysis provided to allow replication by others?

Yes

Reviewer Expertise:

NA

I confirm that I have read this submission and believe that I have an appropriate level of expertise to confirm that it is of an acceptable scientific standard.

# Peer review: Reviewer response for version 2

**DOI:** 10.5256/f1000research.79656.r101239

**Published:** 2021-12-07

**Authors:** A. Abinawanto

**Affiliations:** 1 Department of Biology, Faculty of Mathematics and Natural Sciences, Universitas Indonesia, Depok, Indonesia

This research article provides the information (data) comprehensively. The study was designed appropriately and the method was explained in detail. This research article was also supported by statistical analysis. The conclusion was drawn adequately. The current literature was cited in this article. The authors have sufficiently addressed my previous comments. Accordingly, I strongly recommended this research article be indexed.

Is the work clearly and accurately presented and does it cite the current literature?

Yes

If applicable, is the statistical analysis and its interpretation appropriate?

Yes

Are all the source data underlying the results available to ensure full reproducibility?

Yes

Is the study design appropriate and is the work technically sound?

Yes

Are the conclusions drawn adequately supported by the results?

Yes

Are sufficient details of methods and analysis provided to allow replication by others?

Yes

Reviewer Expertise:

NA

I confirm that I have read this submission and believe that I have an appropriate level of expertise to confirm that it is of an acceptable scientific standard.

# Peer review: Reviewer response for version 1

**DOI:** 10.5256/f1000research.56217.r100290

**Published:** 2021-11-18

**Authors:** A. Abinawanto

**Affiliations:** 1 Department of Biology, Faculty of Mathematics and Natural Sciences, Universitas Indonesia, Depok, Indonesia

The manuscript is quite good, generally. I made some corrections to the manuscript. These include species names being written in italic. In some tables should also add a “plus-minus mark” (±) following the average. In the Abstract, it should be explained the statistic analysis used in this study.

 Methods: The authors should specify the basis of choosing different concentration fish feed of *B. pandurata* as well as 4 and 8 weeks of observation?. The study lacks a specific hypothesis. Reference: #1, it should be translated into English.

Is the work clearly and accurately presented and does it cite the current literature?

Yes

If applicable, is the statistical analysis and its interpretation appropriate?

Yes

Are all the source data underlying the results available to ensure full reproducibility?

Yes

Is the study design appropriate and is the work technically sound?

Yes

Are the conclusions drawn adequately supported by the results?

Yes

Are sufficient details of methods and analysis provided to allow replication by others?

Yes

Reviewer Expertise:

Animal Physiology, Animal Genetics, Cell and Molecular Biology.

I confirm that I have read this submission and believe that I have an appropriate level of expertise to confirm that it is of an acceptable scientific standard, however I have significant reservations, as outlined above.

# Response

**Published:** 2021-11-18

**Authors:** Esti Handayani Hardi

**Affiliations:** Mulawarman University, Indonesia

Thank you for the review of my article, I made some revisions

 :

1. Checking again the italics 
2.  We have added more information and literature to make more clearly about the dose. the dose or concentration was using this research came from the research before (Hardi *et al., *2016;2017;2018;2019).
3.  Revision in discussed chapter with adding the statistic results. 

# Peer review: Reviewer response for version 1

**DOI:** 10.5256/f1000research.56217.r95791

**Published:** 2021-10-22

**Authors:** Angela Mariana Lusiastuti

**Affiliations:** 1 Research Institute for Freshwater Aquaculture and Fisheries Extension (RIFAFE), Bogor, Indonesia

I think this script is good, using herbs as an alternative to chemical drugs. Abstract, Introduction, Methods, and Results are good. The focus of the Discussion should be on BPE as an immunostimulant, not as a prebiotic. If you want to discuss prebiotics, you must include parameters related to the effects of prebiotics. For example prebiotic characteristics, prebiotics activity in the intestinal and other BPE performances as prebiotics such as digestive enzyme activity, short-chain fatty acid, or the diversity of the gut microbiome. If you want to compare research results with other people's research, it's best to use the same BPE or at least have the same active ingredients of BPE. not only compared with herbs that have different criteria.  I think the reason for increased growth and FCR reduction is because of effects from BPE as immunostimulant not from the positive physiological impact that carbohydrates (oligosaccharides) and the essential nutrient pectin produced on the digestive system by reducing glucose absorbance, If you use this reason, you should have a proof that your feed formula contains carbohydrates (oligosaccharides) and the essential nutrient pectin (Table 1).

Is the work clearly and accurately presented and does it cite the current literature?

Yes

If applicable, is the statistical analysis and its interpretation appropriate?

Yes

Are all the source data underlying the results available to ensure full reproducibility?

Yes

Is the study design appropriate and is the work technically sound?

Yes

Are the conclusions drawn adequately supported by the results?

Yes

Are sufficient details of methods and analysis provided to allow replication by others?

Yes

Reviewer Expertise:

Fish Pathology

I confirm that I have read this submission and believe that I have an appropriate level of expertise to confirm that it is of an acceptable scientific standard, however I have significant reservations, as outlined above.

# Response

**Published:** 2021-11-12

**Authors:** Esti Handayani Hardi

**Affiliations:** Mulawarman University, Indonesia

Thank you very much for the revision and response.

 In this research, I want to evaluate the effect of *Boesenbergia pandurata* Application in Goldfish (Cyprinus carpio) specifically on growth, immunity, and resistance to pathogens in general. By looking at the parameters WG, SGR, FCR, Immunological parameters, and SR. At the beginning of this research, separate extracts were made which were then mixed in feed and showed good results. Furthermore, the researchers made a feed formulation by adding BPE so that they could evaluate whether the effect was the same for carp or not. Whether its function as a prebiotic or as a growth stimulator mechanism has not yet been seen, but its effect on goldfish has been seen directly. The discussion presented tried to explore and evaluate several researchers regarding the opportunities for plant extracts as prebiotics. Initial tests showed that BPE showed carbohydrate content (Hardi *et al., *2016).
